# Small Bowel Obstruction Due to Axial Torsion of Meckel’s Diverticulum: A Case Report and Literature Review

**DOI:** 10.7759/cureus.50934

**Published:** 2023-12-22

**Authors:** Hironari Kawai, Nobuo Omura, Tsuyoshi Hirabayashi, Tetsuya Shimada, Hidejiro Kawahara

**Affiliations:** 1 Surgery, Nishisaitama-Chuo National Hospital, Tokorozawa, JPN; 2 Pathology, Nishisaitama-Chuo National Hospital, Tokorozawa, JPN

**Keywords:** meckel’s diverticulum, internal hernia, long intestinal tube, nasogastric tube (ngt), mesodiverticular band

## Abstract

Meckel’s diverticulum (MD) is a commonly encountered congenital gastrointestinal abnormality. Although the frequency of MD-related complications such as inflammation or bleeding is relatively high, small bowel obstruction induced by axial torsion of the MD is rare. Therefore, we herein report such a case along with a review of the literature. A 34-year-old female with right lower quadrant pain, nausea, and vomiting was admitted to our hospital with the diagnosis of adhesive small bowel obstruction due to a cesarean section performed five years previously. A long intestinal tube was placed, and the patient’s clinical symptoms and X-ray findings showed relief of the small bowel obstruction. However, she developed severe right lower quadrant pain after contrast examination through the long intestinal tube despite the fact that the contrast agent had smoothly reached the terminal ileum. Blood tests and enhanced computed tomography (CT) showed a remarkable elevation of inflammatory markers, the appearance of ascites, and closed-loop-like and abscess-like appearances near the site of the caliber change. With a diagnosis of internal hernia, the patient underwent emergency laparotomy by means of a midline incision. Purulent ascites was observed within the abdominal cavity. Small bowel obstruction caused by a single band was observed in the right lower quadrant. Further exploration revealed an inflammatory MD with neck torsion and a mesodiverticular band (MDB). Simple mesodiverticular band resection by electrocautery and diverticulectomy by linear stapler were performed. The postoperative course was uneventful, and the patient was discharged on postoperative day 7. In the case of juvenile-onset small bowel obstruction, axial torsion of the MD should be considered as a differential diagnosis. Herein, we report such a difficult diagnostic case and the first English literature review of small bowel obstruction due to axial torsion of the MD.

## Introduction

Meckel’s diverticulum (MD) is the most common congenital gastrointestinal abnormality, affecting 2% of the population. MD is caused by the incomplete obliteration of the vitelline duct, leading to the formation of an enterocyst or a mesodiverticular band (MDB) [1]. The lifetime risk of complications such as gastrointestinal bleeding, inflammation, or obstruction in patients with MD is only 4% [2]. Volvulus, intussusception, and incarcerated hernias are considered the main causes of MD-induced small bowel obstruction. Among these causal factors, axial torsion of the MD is a rare complication of small bowel obstruction. We herein report a case of difficult diagnosis of small bowel obstruction due to axial torsion of the MD.

## Case presentation

**Figure 1:**
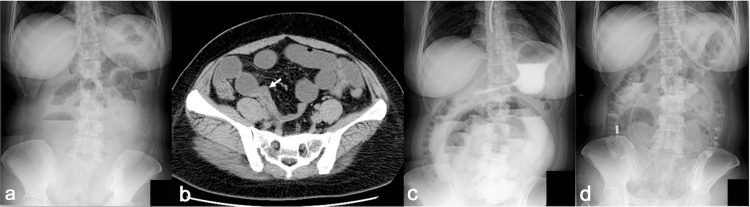
(A) Abdominal X-ray image (standing position) on day 0. (B) Plain CT image at the site of caliber change (arrow). (C) Abdominal X-ray image (standing position) three hours after WSC injection through the nasogastric tube on day 3, showing multiple air-fluid levels. (D) Abdominal X-ray image (standing position) two days after long intestinal tube placement. CT, computed tomography; WSC, water-soluble contrast

 A 34-year-old female with right lower quadrant pain, nausea, and vomiting was admitted to our hospital with the diagnosis of adhesive small bowel obstruction due to a cesarean section performed five years previously. She had experienced no similar episodes in the past. Laboratory tests at admission showed a slight elevation of inflammatory markers (white blood cell count, 11,400/µL; C-reactive protein, 0.50 mg/dL). Abdominal X-ray examination showed multiple air-fluid levels (Figure 1A), and plain computed tomography (CT) revealed a caliber change in the right lower quadrant and distention of the proximal small intestine, indicating small bowel obstruction (Figure 1B). There were no signs of peritoneal irritation or ascites by CT. Nasogastric tube placement and fluid replacement were initiated as the primary treatment on day 0 of admission. The patient’s clinical symptoms and multiple air-fluid levels on X-ray examination had diminished by day 3. Therefore, hyperosmolar water-soluble contrast (WSC) was administered through the nasogastric tube for the diagnosis and management of adhesive small bowel obstruction. However, the patient developed abdominal distension and nausea on day 4. X-ray examination revealed multiple air-fluid levels and a recurrence of the small intestinal distension (Figure 1C). Because conservative treatment by the nasogastric tube was challenging, a long intestinal tube was placed on day 5. The patient’s clinical symptoms and X-ray findings resolved, indicating relief of the small bowel obstruction (Figure 1D).

**Figure 2:**
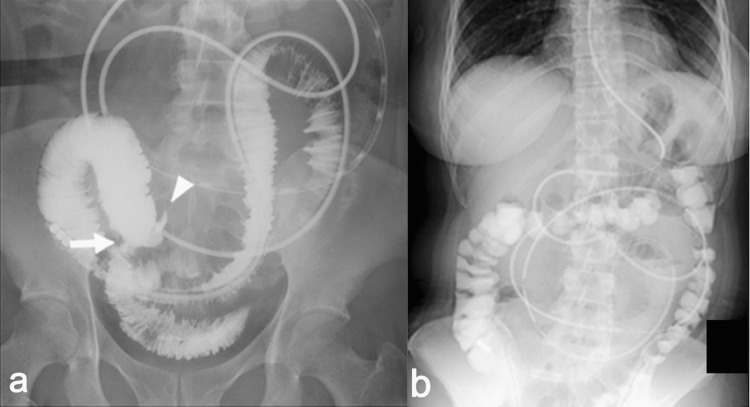
(A) Fluoroscopic X-ray image of WSC injection through the long intestinal tube. Caliber change (arrow) and beak sign-like appearance (arrowhead) were observed. (B) Abdominal X-ray image (standing position) two hours after fluoroscopic X-ray examination showing no WSC within the small intestine. WSC: water-soluble contrast

Laboratory tests on day 5 also showed no abnormal findings. Therefore, a contrast examination using WSC through the long intestinal tube was performed on day 7. The WSC smoothly reached the terminal ileum, although the caliber change in the right lower quadrant persisted (Figure 2A). After the contrast examination, the patient developed severe right lower quadrant pain at night despite the fact that contrast-induced diarrhea was observed and abdominal X-ray examination two hours after fluoroscopic examination showed no residual WSC within the small intestine (Figure 2B).

**Figure 3:**
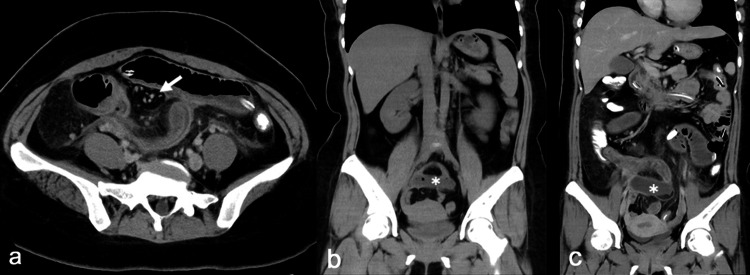
(A) Enhanced CT on day 8 showing the persistent caliber change in the right lower quadrant (arrow). (B) Plain CT at admission and (C) enhanced CT on day 8 showing an abscess-like appearance (asterisk), which was retrospectively confirmed as MD. CT, computed tomography; MD, Meckel’s diverticulum

The severe abdominal pain persisted, and a high fever (38.6℃) developed; therefore, blood tests and enhanced CT were performed on day 8. Laboratory examination showed elevation of the white blood cell count (23,700/µL) and C-reactive protein concentration (19.6 mg/dL). Enhanced CT showed persistent caliber change in the right lower quadrant (Figure 3A). Ascites had also developed, and an abscess-like appearance (retrospectively observed on plain CT at admission as shown in Figure 3B) was observed near the site of the caliber change (Figure 3C).

**Figure 4:**
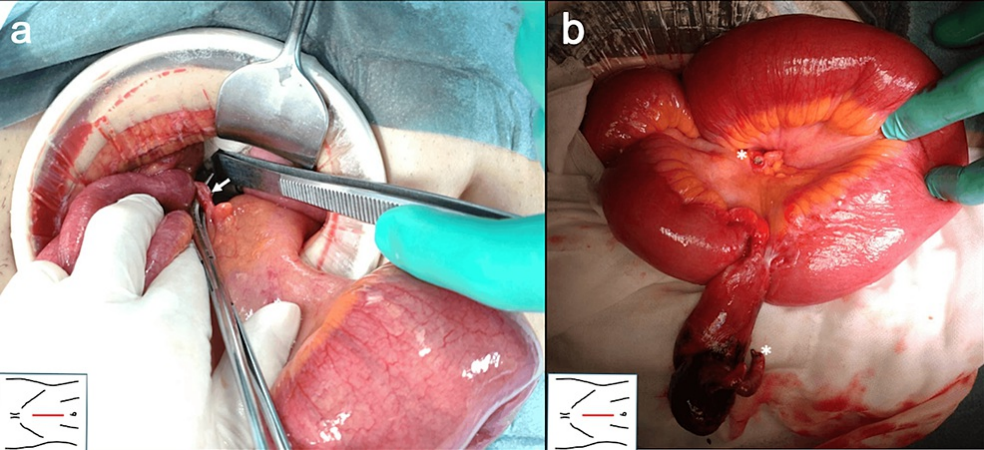
(A) Before and (B) after the dissection of the MDB. The arrow indicates the MDB. The asterisk indicates the dissection point of the MDB. MDB: mesodiverticular band

With a diagnosis of internal hernia, the patient underwent elective laparotomy with a midline incision. Purulent ascites was observed within the abdominal cavity. Consistent with the CT findings, closed-loop obstruction by a single band was observed in the right lower quadrant (Figure 4A). Further exploration revealed an inflammatory MD with neck torsion and an MDB located 70 cm proximal to the terminal ileum (Figure 4B). The small bowel obstruction was released by MDB dissection. Because a necrotic change of the small intestine was not observed, simple MDB resection by electrocautery and diverticulectomy along with a minor axis of the small intestine to prevent the stenosis by a linear stapler was performed. Diverticulectomy was performed including the boundary with the ileum for the prevention of ulceration due to ectopic gastric mucosa.

**Figure 5:**
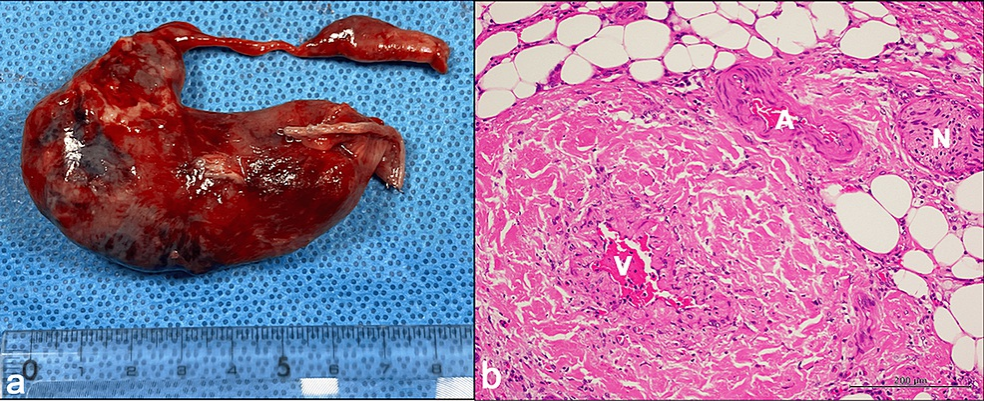
(A) Gross findings of the resected MD with MDB. (B) Pathological findings of the MDB. Large vasculature was observed within the band, consistent with an MDB. MD, Meckel’s diverticulum; MDB, mesodiverticular band A, artery; V, vein; N, nerve

Postoperative gross examination showed an inflammatory MD (7 × 2 cm) with a band (5 cm in length) at the tip of the diverticulum (Figure 5A). Histopathological examination revealed inflammatory erosive and ulcerative mucosa within the MD. The proper evaluation of the presence of ectopic tissue was difficult because of the extensive mucosal erosion and ulceration. Relatively large vasculature was observed within the band, consistent with an MDB (Figure 5B). The intraoperative images in the present case clearly indicate that arterial blood supply to the MD arises from the ileocolic branches of the superior mesenteric artery (Figures 4B, 5B). The postoperative course was uneventful, and the patient was discharged on postoperative day 7.

## Discussion

In the present case, we have obtained the following novel and instructive findings: 1) contrast examination may have triggered the complete axial torsion of the MD, which is rare and an instructive clinical course; 2) there are no previous fluoroscopic images of axial torsion of the MD, making the image in the present case the first; 3) the intraoperative images clearly indicate that arterial blood supply to the MD arises from the ileocolic branches of the superior mesenteric artery, which is impactful and easy to understand; and 4) this is a first review of small bowel obstruction due to axial torsion of Meckel’s diverticulum in the English literature. These findings are noteworthy and suggestive for practitioners.

MD was first described by Fabricius Hildanus in 1598 and later named after Johann Friedrich Meckel, a German anatomist who first recognized its developmental origin in 1809. Small bowel obstruction due to volvulus, intussusception, or incarcerated hernia with MD is relatively common in the clinical setting. However, small bowel obstruction induced by axial torsion of the MD and MDB is rare. Eighteen previous reports in the English-language literature describing small bowel obstruction by axial torsion of the MD are listed in Table 1 (according to a PubMed search for “Meckel’s diverticulum,” “axial torsion or torsion,” and “small bowel obstruction or obstruction”) [1,3-19]. The sex ratio (male, n = 12; female, n = 5; not available {NA}, n = 1) is consistent with a previous report indicating that symptomatic MD is more common in male than female patients [20]. According to that previous report, an age of <50 years, male sex, and a diverticulum length of >2 cm are most commonly associated with symptomatic MD [20]. Fifteen of the 18 patients summarized in Table 1 were <50 years of age; therefore, the involvement of the MD should be considered as a differential diagnosis of small bowel obstruction in young patients. None of these cases were preoperatively diagnosed as torsion of MD, implying the difficulty of accurate diagnosis of axial torsion of the MD. With respect to operative methods, ileal resection or diverticulectomy was performed (Table 1).

**Table 1:** Eighteen previous cases and the present case of a small bowel obstruction with axial torsion of Meckel’s diverticulum. ＋: had a past history of previous surgery; −: no past history of previous surgery NA, not available; M, male; F, female

Number	Year	Reference	Age	Sex	Chief complaint	Previous surgery	Preoperative diagnosis	Duration from consultation to surgery (days)	Distance from ileal end (cm)	Size (cm)	Operation
1	1998	Malhotra et al. [3]	54	M	Right lower quadrant abdominal pain/nausea	＋	NA (exploratory laparotomy)	NA	NA	NA	Open ileal resection
2	2006	Limas et al. [4]	6	M	Abdominal pain/nausea/fever	NA	Acute appendicitis	0	50	16 × 4	Open diverticulectomy
3	2011	Cartanese et al. [5]	42	M	Lower quadrant and suprapubic pain/vomiting	−	Suspected appendicitis	0	50	11 × 1.5	Open diverticulectomy
4	2013	Sasikumar et al. [6]	26	M	Abdominal pain/distention/vomiting	−	Appendicular mucocele/acute intestinal obstruction	0	NA	NA	Open ileal resection
5	2014	Murruste et al. [7]	41	M	Abdominal pain/distention/nausea	−	NA	NA	50	12 × 14	Open ileal resection
6	2015	Tenreiro et al. [8]	18	M	Abdominal pain/distention/vomiting	−	Small bowel obstruction	2	50	12 × 2	Open ileal resection
7	2015	Hadeed et al. [9]	29	F	Right lower quadrant abdominal pain	NA	Small bowel obstruction with closed loop	NA	30	NA	Lap diverticulectomy
8	2015	Xanthis et al. [10]	52	M	Right-sided abdominal pain/vomiting	NA	Strangulating bowel obstruction	NA	NA	NA	Lap diverticulectomy
9	2015	Ren et al. [11]	32	M	Abdominal pain/fever	−	NA (exploratory laparotomy)	NA	90	12 × 5	Open ileal resection
10	2015	Seshadri et al. [12]	65	M	Right lower quadrant abdominal pain	−	NA (exploratory laparotomy)	NA	60	8	Open diverticulectomy
11	2016	Luu et al. [13]	34	NA	Abdominal pain/nausea/vomiting	−	Small bowel obstruction	0	40	17	Open ileal resection
12	2016	Yıldız et al. [14]	21	F	Abdominal pain/vomiting/fever	−	Acute abdomen	NA	45	12 × 3	Open diverticulectomy
13	2019	Nagata et al. [15]	31	F	Epigastric pain/nausea/vomiting	＋	Hyperemesis gravidarum	1	2	11 × 8	Open ileal resection
14	2020	Ajmal et al. [16]	25	M	Abdominal pain/nausea/vomiting	−	Small bowel obstruction	0	50	NA	Open ileal resection
15	2021	Jha et al. [17]	13	F	Abdominal pain/distention/vomiting	−	Acute intestinal obstruction	NA	30	10 × 2	Open ileal resection
16	2022	Bejiga and Ahmed [18]	20	M	Abdominal pain/vomiting	−	Complicated appendicitis	0	60	8	Open ileal resection
17	2022	Munasinghe et al. [19]	20	M	Left lower abdominal pain/vomiting	−	NA (exploratory laparotomy)	0	45	25 × 2	Open ileal resection
18	2023	Kafshgari et al. [1]	5	F	Abdominal pain/vomiting	−	Obstructive intestinal volvulus	0	NA	NA	Open ileal resection
19	2023	Present study	34	F	Right lower quadrant abdominal pain/nausea/vomiting	＋	Strangulating bowel obstruction	8	70	8 × 2	Open diverticulectomy

Regarding the timing of the operation, eight of the 18 patients underwent an emergency operation immediately after visiting the outpatient clinic (data are not available for the eight patients). Compared with these patients, our patient underwent surgery eight days after the first visit, probably because of the temporary relief of the small bowel obstruction and misdiagnosis at admission. There are two plausible reasons for the misdiagnosis at admission in the present case. First, the patient’s surgical history might have led to the misdiagnosis of adhesive small bowel obstruction. Notably, only two of the 18 previously described patients had a surgical history (Table 1). Second, with respect to the imaging findings, it was difficult to distinguish the MD from the small intestine because the MD was dilated to the same degree as the small intestine. Although the radiologist read the images in advance, the preoperative diagnosis was adhesive small bowel obstruction or internal hernia, indicating the difficulty of the accurate diagnosis of axial torsion of the MD. However, the discontinuity of the lumen, which is the characteristic CT finding of the MD, was confirmed in retrospect [2]. These factors might have led to delayed diagnosis. In the present case, the decompression of the small intestine by a long intestinal tube was successfully performed in advance; therefore, exploratory laparoscopy might have been an option for the diagnosis and surgical treatment.

Our patient’s clinical course dramatically changed after the contrast examination using WSC through the long intestinal tube. Her clinical findings were relieved by long intestinal tube placement, but severe abdominal pain occurred after contrast examination. The radical increase of intraluminal pressure induced by the contrast examination might have provoked MD sub-torsion to progress to complete torsion, followed by inflammatory diverticulitis, fixation to the retroperitoneum, incarceration of the distal ileum into the MDB, and finally small bowel strangulation. The fluoroscopic X-ray findings in Figure 2A showed a beak sign-like appearance and no inflow of WSC into the MD probably because of the torsion of the MD, whereas WSC enters the MD under normal conditions [2]. To the best of our knowledge, there are no previous fluoroscopic images of axial torsion of the MD, making the image in the present case the first. Additionally, the location of the MD had moved approximately 2 cm toward the cranial side after the contrast examination (Figure 3B, 3C). This CT finding partially corroborates the abovementioned presumed pathophysiology in the present case. The contrast examination might have triggered the MD torsion and small bowel obstruction, although this is difficult to validate.

In summary, in cases of juvenile-onset small bowel obstruction, the consideration of MD involvement is the most important factor in attaining an accurate preoperative diagnosis and may lead to the detection of axial torsion of the MD by imaging modalities.

## Conclusions

We have reported a case and a first literature review of small bowel obstruction due to axial torsion of the MD, which could be worsened by contrast examination. Axial torsion of the MD should be considered as a differential diagnosis in patients with juvenile-onset small bowel obstruction to attain an accurate preoperative diagnosis.
